# Psychophysical Evaluation of the Olfactory Function: European Multicenter Study on 774 COVID-19 Patients

**DOI:** 10.3390/pathogens10010062

**Published:** 2021-01-12

**Authors:** Luigi Angelo Vaira, Jerome R. Lechien, Mohamad Khalife, Marzia Petrocelli, Stephane Hans, Lea Distinguin, Giovanni Salzano, Marco Cucurullo, Piero Doneddu, Francesco Antonio Salzano, Federico Biglioli, Fabrice Journe, Andrea Fausto Piana, Giacomo De Riu, Sven Saussez

**Affiliations:** 1 COVID-19 Task Force of the Young-Otolaryngologists of the International Federations of Oto-rhino-laryngological Societies (YO-IFOS), F92150 Paris, France; jerome.lechien@umons.ac.be (J.R.L.); mohamad.khalife@epicura.be (M.K.); prhans.foch@gmail.com (S.H.); lea.distiguin@hotmail.fr (L.D.); gderiu@uniss.it (G.D.R.); 2 Maxillofacial Surgery Operative Unit, University Hospital of Sassari, 07100 Sassari, Italy; piero.doneddu@gmail.com; 3 Biomedical Science Department, PhD School of Biomedical Science, University of Sassari, 07100 Sassari, Italy; piana@uniss.it; 4 Department of Human and Experimental Oncology, Faculty of Medicine UMONS Research Institute for Health Sciences and Technology, University of Mons (UMons), B7000 Mons, Belgium; fabrice.journe@umons.ac.be; 5 Department of Otolaryngology-Head & Neck Surgery, Foch Hospital, School of Medicine, UFR Simone Veil, Université Versailles Saint-Quentin-en-Yvelines (Paris Saclay University), F92150 Paris, France; 6 Department of Otorhinolaryngology and Head and Neck Surgery, CHU de Bruxelles, CHU Saint-Pierre, School of Medicine, Université Libre de Bruxelles, B1000 Brussels, Belgium; 7 Department of Otolaryngology-Head & Neck Surgery, EpiCURA Hospital, B 7000 Baudour, Belgium; 8 Maxillofacial Surgery Operative Unit, Bellaria and Maggiore Hospital, 40100 Bologna, Italy; marzia.petrocelli@ausl.bologna.it; 9 Maxillofacial Surgery Operative Unit, University Hospital of Naples “Federico II”, 80131 Naples, Italy; giovannisalzanomd@gmail.com; 10 Maxillofacial Surgery Department, San Paolo Hospital, ASST Santi Paolo e Carlo, University of Milan, 20142 Milan, Italy; mar.cucurullo@gmail.com (M.C.); federico.biglioli@unimi.it (F.B.); 11 Otorhinolaryngology Operative Unit, Department of Medicine, Surgery and Dentistry, “Scuola Medica Salernitana”, University of Salerno, 84081 Baronissi (Salerno), Italy; frsalzano@unisa.it; 12 Clinical Epidemiology and Medical Statistics Unit, Department of Medical, Surgical and Experimental Sciences, University of Sassari, 07100 Sassari, Italy

**Keywords:** COVID-19, SARS-CoV-2, anosmia, hyposmia, olfactory dysfunction, smell, olfaction, parosmia

## Abstract

Background: The objective evaluation of the olfactory function of coronavirus disease 2019 patients is difficult because of logistical and operator-safety problems. For this reason, in the literature, the data obtained from psychophysical tests are few and based on small case series. Methods: A multicenter, cohort study conducted in seven European hospitals between March 22 and August 20, 2020. The Sniffin-Sticks test and the Connecticut Chemosensory Clinical Research Center orthonasal olfaction test were used to objectively evaluate the olfactory function. Results: This study included 774 patients, of these 481 (62.1%) presented olfactory dysfunction (OD): 280 were hyposmic and 201 were anosmic. There was a significant difference between self-reported anosmia/hyposmia and psychophysical test results (*p* = 0.006). Patients with gastroesophageal disorders reported a significantly higher probability of presenting hyposmia (OR 1.86; *p* = 0.015) and anosmia (OR 2.425; *p* < 0.001). Fever, chest pain, and phlegm significantly increased the likelihood of having hyposmia but not anosmia or an olfactory disturbance. In contrast, patients with dyspnea, dysphonia, and severe-to-critical COVID-19 were significantly more likely to have no anosmia, while these symptoms had no effect on the risk of developing hyposmia or an OD. Conclusions: Psychophysical assessment represents a significantly more accurate assessment tool for olfactory function than patient self-reported clinical outcomes. Olfactory disturbances appear to be largely independent from the epidemiological and clinical characteristics of the patients. The non-association with rhinitis symptoms and the high prevalence as a presenting symptom make olfactory disturbances an important symptom in the differential diagnosis between COVID-19 and common flu.

## 1. Introduction

Olfactory dysfunctions (OD) represent one of the most frequent and specific symptoms of coronavirus disease 2019 (COVID-19), affecting approximately 65–70% of patients [1,2,3,4,5,6]. COVID-19 patients may present a wide range of OD: altered perception of odors (parosmia), perception of odors even if they are not present (phantosmia), or partial (hyposmia) or total (anosmia) reduction of the olfactory function [2,3,4,7]. The pathogenesis of OD in COVID-19 has not yet been elucidated [8], but it is thought to be related to neuroinvasion [9,10] or, more likely, to inflammatory phenomena at the level of the olfactory epithelium [11,12,13].

The majority of recent studies about OD frequency in COVID-19 patients are based on the analysis of medical records or on patient-reported outcomes. The realization of objective olfactory assessments of these patients was difficult during the pandemic as a result of the sanitary recommendation of avoiding the endonasal examination, the home-management of many patients in quarantine, and therefore, the inability to perform psychophysical testing [14]. To date, only a few objective studies have been conducted on small case series, thus reliable conclusions are limited [15,16,17,18,19,20,21,22,23,24]. However, the objective olfactory assessment with validated, repeatable, and standardized tests remains crucial to confirm, characterize, and follow the OD over time. 

The aim of this European multicenter study was to objectively evaluate the olfactory functions of 774 COVID-19 patients within the first weeks of diagnosis with validated psychophysical tests. 

## 2. Materials and Methods

The study was conducted in the following European hospitals: EpiCURA Hornu (Belgium); Foch Hospital (Paris, France); and University Hospital of Sassari (Italy), University Hospital of Milan (Italy), University Hospital of Salerno (Italy), and Bellaria-Maggiore Hospital of Bologna (Italy), between March 22 and August 20, 2020. To be enrolled, patients had to meet the following inclusion criteria: adult over 18 years of age; severe acute respiratory syndrome coronavirus 2 (SARS-CoV-2) infection confirmed after a nasopharyngeal swab (RT-PCR); and a COVID-19 clinical onset (or a positive swab in asymptomatic patients) for less than 15 days. Patients with a negative RT-PCR were given a serological test (Zentech, University of Liege Lab, Liege, Belgium) to determine whether or not they had been exposed to SARS-Cov-2.

Patients were excluded if they presented a history of previous olfactory dysfunction, chronic and allergic rhinosinusitis, nasal or olfactory cleft surgery, radiotherapy, or trauma to the oral and nasal cavities.

### 2.1. Demographic, Epidemiological, and Clinical Outcomes

The following demographic and epidemiological data were collected with a standardized online questionnaire: gender; age; ethnicity; smoking and comorbidities (i.e., heart, respiratory, kidney, liver, neurological, autoimmune diseases, diabetes, hypertension, hypothyroidism, chronic and allergic rhinosinusitis, gastroesophageal reflux, depression, and untreated cancer) [25]. Note that the majority of patients filled out the questionnaire themselves; however, physicians completed the questionnaire after an anamnesis for the hospitalized patients who were not able to electronically complete the evaluations. The survey included specific questions regarding the loss of smell, its duration, or the presence of qualitative alterations in olfactory perception (e.g., parosmia and phantosmia). The evaluation of the self-reported olfactory function was based on the data collected through this questionnaire. From a clinical standpoint, general symptoms were assessed as present or absent. Otolaryngological symptoms were rated with a 5-point scale ranging from 0 (absent) to 4 (very severe symptoms) through items from the smell and taste component of the National Health and Nutrition Examination Survey (NHNES) [26]. Following the criteria proposed by Tian et al. [27], subjects were divided into four groups of clinical severity: mild-to-moderate and severe-to-critical.

### 2.2. Objective Olfactory Outcomes 

Sniffin-Sticks identification tests (Medisense, Groningen, Netherlands) were used to assess the objective olfactory function in patients evaluated at EpiCURA Hornu and Foch Hospitals. The Sniffin-Sticks test is a validated psychophysical olfactory test using 16 smell pens. Each pen was presented to individual who had to choose the adequate smell between four given options [28]. Depending on the score obtained, patients were classified into the following categories: normosmia (score between 12 and 16); hyposmia (score between 9 and 11); and anosmia (score < 9).

In patients enrolled in the four Italian hospitals, the olfactory function assessment was carried out by means of the Connecticut Chemosensory Clinical Research Center orthonasal olfaction test (CCCRC) [29]. The CCCRC test includes a N-butanol threshold assessment and a 10-items odor identification test using common odors. Like the Sniffin-Sticks tests, the CCCRC test classifies the olfactory function of patients as normal (score between 90 and 100), hyposmia (score between 20 and 80), and anosmia (score between 0 and 10). 

### 2.3. Statistical Analyses 

Statistical analyses were performed using the Statistical Package for the Social Sciences for Windows version 26.0 (IBM Corp, Armonk, NY, USA). Categorical variables are reported in numerals and percentages of the total. Descriptive statistics for quantitative variables are given as the mean ± standard deviation (SD). For the purposes of the statistical analysis, the patients were classified into three categories of olfactory function according to the psychophysical scores obtained: normal, hyposmic, and anosmic. X^2^-square test was performed to evaluate the effects of each clinical feature on the proportion of normal, hyposmic, and anosmic patients. A cross-tabulation analysis was then performed to assess the statistical correlation between clinical and olfactory findings. To avoid type II statistical errors, the minimum size of the subgroups was determined using G*power 3.1 (Heinrich Heine University Dusseldorf, Dusseldorf, Germany). The calculation was based on total sample size 774 subjects, 0.5 Cohen’s D, 95% power, and 5% margin of error resulting in a minimum subgroup sample size of 59 patients. Otolaryngological symptoms were therefore considered as present or absent for the purposes of statistical analysis, as the subgroups did not reach the minimum number of subjects. The level of statistical significance was set at *p* < 0.05 with a 95% confidence interval.

## 3. Results

A total of 774 patients met the inclusion criteria. Among them, 231 and 543 were objectively evaluated by means of Sniffin-Sticks and CCCRC tests, respectively. The demographic and clinical characteristics of patients are summarized in Table 1. Furthermore, 55.6% of the patients were females. The mean age was 46.3 ± 14 years old. Hypertension was the most common comorbidity (19.2%), followed by gastroesophageal disorders (11.4%), and diabetes (10.8%). Overall, 46.4% of patients had at least one comorbidity.

### 3.1. General and Otolaryngological Outcomes

One hundred and eleven patients (14.3%) were completely asymptomatic at the time of evaluation, and the diagnosis was made after a routine control swab. One hundred four patients were found to have severe-to-critical COVID-19 (13.4%) while 519 had mild-to-moderate forms (67.1%). The clinical severity of COVID-19 was determined in 734 patients.

The most reported symptoms were asthenia (61.4%), headache (45%), anorexia (39.5%), and cough (39.1%) (Table 1). The prevalence and severity of otolaryngological symptoms according to NHNES were performed in 636 patients. A framework summary of the results obtained is reported in Table 2. Nasal obstruction (48.3%), rhinorrhea (45.6%), and postnasal drip (37.3%) were the most common otolaryngological symptoms in COVID-19 patients.

### 3.2. Subjective and Objective Olfactory Dysfunctions

Four hundred and fifty-one patients (58.4%) self-reported an olfactory disturbance during the COVID-19 clinical course (Table 3). 

Hyposmia affected 23.6% of patients, while anosmia was found in 29.7%. Phantosmia and parosmia affected 13.6% and 26.4% of patients, respectively. Olfactory disorder was one of the presenting symptoms of COVID-19 in 29.3% of cases and the only one in 94 patients (12.1%). Regarding objective tests, 481 patients (62.1%) presented olfactory dysfunction: 280 were hyposmic and 201 were completely anosmic (Table 3). There was a significant difference between self-reported anosmia/hyposmia and psychophysical test results (χ^2^ statistic 10.360; *p* = 0.006). The mean olfactory score was 10.4 ± 3.7 for patients undergoing the Sniffin-Sticks test (test range 0–16) and 57.8 ± 36.8 for those evaluated with the CCCRC test (test range 0–100).

**Figure 1:**
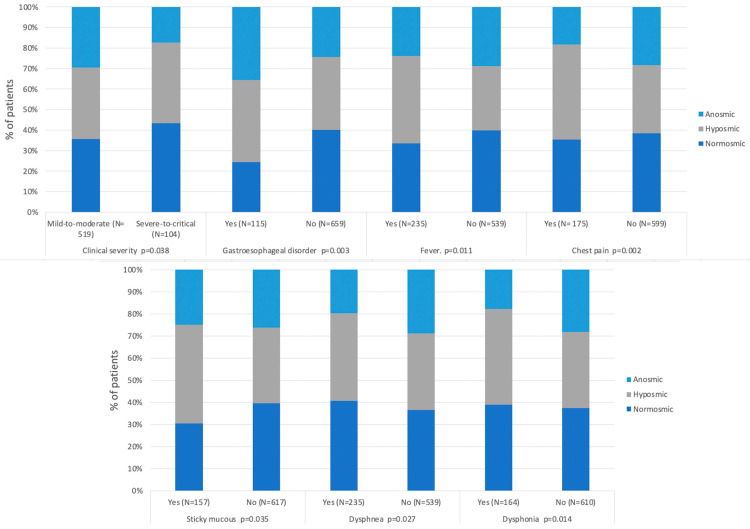
Olfactory and clinical outcome significant correlation.

The correlation analysis between clinical outcomes and the proportion of normal, hyposmic, and anosmic patients is summarized in Supplementary Table S1 and Figure 1. 

Most of the parameters assessed did not show significant correlations with the OD. In particular, there were no correlations between OD severity and the presence of nasal rhinitis symptoms such as nasal obstruction (*p* = 0.184), rhinorrhea (*p* = 0.279), and postnasal drip (*p* = 0.287).

**Figure 2:**
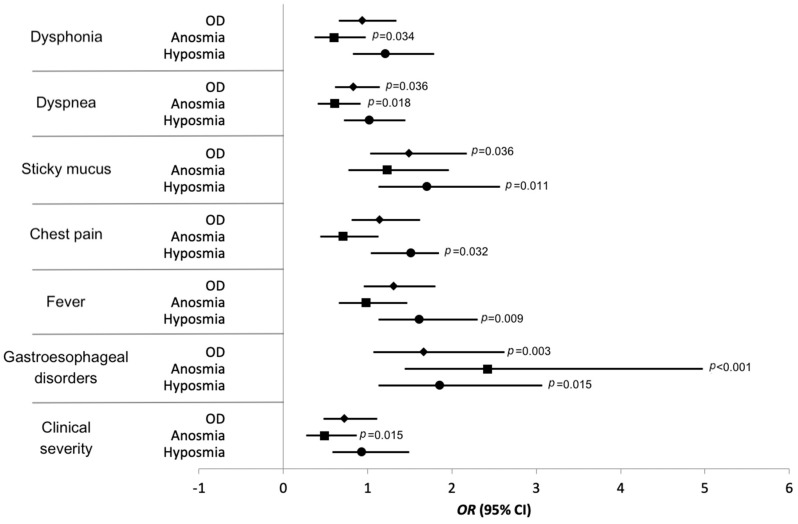
Cross-tab analysis results. Abbreviations: OD: olfactory dysfunction, e.g., anosmia or hyposmia; OR: odds ratio; CI: confidence interval.

Patients with gastroesophageal disorders (i.e., ulcer, gastroesophageal reflux, and laryngopharyngeal reflux) reported a significantly higher probability of presenting hyposmia (odds ratio 1.86; 95% confidence interval 1.127–3.072; *p* = 0.015) and anosmia (odds ratio 2.425; 95% confidence interval 1.443–4.975; *p* < 0.001) on objective tests. Fever, chest pain, and phlegm significantly increased the likelihood of having hyposmia, but not anosmia or an olfactory disturbance (e.g., anosmia or hyposmia) (Figure 2, Supplementary Table S2). In contrast, patients with dyspnea and dysphonia were significantly more likely to have no anosmia, while these symptoms had no effect on the risk of developing hyposmia or an olfactory disorder (Figure 2, Supplementary Table S2). 

Overall, patients with severe-to-critical COVID-19 demonstrated a significantly lower likelihood of having anosmia (odds ratio 0.484; 95% confidence interval 0.269–0.87; *p* = 0.015), the severity of the picture had no significant statistical correlation with the risk of developing hyposmia or olfactory disturbances. 

## 4. Discussion

Psychophysical tests are currently the clinical gold standard for the evaluation of olfactory function allowing the quantification of the potential dysfunction [30]. However, the realization of these tests is still difficult in infected patients regarding safety problems for operators and patient quarantine. Furthermore, some of the most used tests, such as the University of Pennsylvania Smell Identification Tests, were available in very limited quantities on the European market in the first months of the pandemic. For these reasons, most of the studies published so far are based on the analysis of medical records or on patient interviews, often carried out retrospectively and after an important delay regarding the onset of symptoms of patients. These studies can, therefore, underestimate the frequency of olfactory disturbances due to recall-bias or a poor medical history collected at the time of the evaluation, and in any case, they do not allow an effective quantification of the severity of the disturbance [31]. This point is strengthened regarding the substantial rate of short-term recovery exhibited in the initial multicenter European study identifying the loss of smell as a COVID-19 symptom [2]. On the other hand, studies based on psychophysical tests in COVID-19 patients are few and mostly based on small series or carried out with new tests adapted to emergency situations. Moreover, in most cases they objectively evaluate patients at a great distance from the onset of symptoms, and for this reason, they can only fully grasp the disorder in the phase of resolution or when fully recovered.

The main strength of this study is the objective evaluation of a very large case series at a short distance from the clinical onset. The results obtained should therefore provide a faithful estimate of olfactory function in the early stages of COVID-19. The results of this study showed that there would be a mismatch between the self-reported loss of smell and the findings of psychophysical olfactory evaluations. Thus, among the 323 patients who did not self-report an olfactory dysfunction, 47 had hyposmia on objective tests. On the other hand, four patients who presented normal function on tests had self-reported the presence of hyposmia. Moreover, among the 230 patients who reported the presence of anosmia, 43 presented severe hyposmia on psychophysical tests, and 18 of those who self-reported hyposmia had anosmia. Overall, the frequency of olfactory disorders self-reported by patients and that detected by psychophysical tests was statistically significant. Obviously, a careful selection of the exclusion criteria is still essential in order to minimize the bias introduced by the inclusion of patients with previous olfactory disorders or evaluated in too advanced stages of the disease (i.e., when the disorder is already partially or totally recovered). However, these results make us understand how the exclusive use of self-reported data can introduce enormous bias if used to evaluate the prevalence, the diagnostic and prognostic value, or the recovery of the olfactory disorder in COVID-19 patients. 

Some authors found significant associations between self-reported olfactory disorders and female gender [2,7,32,33,34,35], younger age [32,33,36,37,38], smoker [39] or no-smoker [33] habit, hypertension [15], diabetes [18], depression [40], or symptoms such as fever [2] and nasal obstruction [41]. 

It has been hypothesized that the higher prevalence in women and those of a younger age, reported by some authors, could be related to a more intense inflammatory response that occurs in these patients after the spread of the virus in the olfactory epithelium [2,42,43,44,45]. Note that these authors did not assess the olfactory function with objective testing, rendering the conclusion unreliable. However, our study found no significant differences between the olfactory scores reported by subgroups of patients selected on the basis of gender and age. As regards comorbidities, the only significant relationship was found between the severity of the olfactory disturbance and the presence of gastroesophageal reflux. Chemosensory disorders in gastroesophageal and laryngopharyngeal reflux disease have already been suggested [46] making it possible that laryngopharyngeal reflux may worsen the olfactory perception even in COVID-19. 

In recent months, the prognostic value of olfactory dysfunctions has been the subject of heated debate. Several authors found a significantly lower prevalence of olfactory disorders in hospitalized patients or in severe COVID-19 [18,33,47,48,49]. However, these studies are based on self-reported or clinical record data, which may be subject to inaccuracy due to recall-bias or incomplete medical history. Other authors found no association between olfactory disturbances and the clinical severity of COVID-19 [16,19,20,22,37,38,50,51]. In our study, anosmia was a statistically significant protective factor both for the presence of dyspnea (one of the symptoms most associated with pulmonary deterioration) and for the development of severe-to-critical forms of COVID-19. Interestingly, both dyspnea and severe COVID-19 were not associated with a lower prevalence of hyposmia or an olfactory disorder in general.

The lack of significant associations between olfactory scores otolaryngological symptoms was found in many previous studies [1,2,3,5,6,7,8,9,10,11,12,13,14,15,16,17,18,19,20,52], supporting the hypothesis that COVID-19 olfactory disturbances are not related to rhinitis (i.e., nasal obstruction, rhinorrhea, post-nasal drip) but are probably neurological. This peculiarity, combined with the fact that this chemosensitive disorder represents the presenting symptom of COVID-19 in 29.3% of cases [Table 3], makes olfactory dysfunctions the key symptom in the differential diagnosis between COVID-19 and common flu [36,52,53,54]. The ability to identify among patients with flu symptoms from those who have a high suspicion of COVID-19 represents one of the great challenges facing us in the near future. Given the high prognostic value of olfactory disorders, psychophysical tests and, in particular, the evaluation of the olfactory threshold could be useful for patients with non-specific flu or common cold symptoms.

A possible limitation of this study could be represented by the use of two different psychophysical tests. The different scoring scale of the two tests did not allow us to carry out an overall logistic regression analysis. However, the two tests are widely validated and standardized. Furthermore, the Sniffin-Sticks test was validated for the first time by means of the CCCRC test [55]. For these reasons, it was possible to evaluate the patients overall, dividing them by clinical classes of olfactory dysfunction.

## 5. Conclusions

Psychophysical assessment represents a significantly more accurate assessment tool for the olfactory function than patient self-reported clinical outcomes. On the basis of the objective scores found in this study, olfactory disturbances appear to be largely independent from the epidemiological and clinical characteristics of the patients. The non-association with rhinitis symptoms and the high prevalence as a presenting symptom make olfactory disturbances an interesting symptom in the differential diagnosis between COVID-19 and common flu.

## Figures and Tables

**Table 1:** Patient characteristics.

Characteristics	Patients (N = 774)
Age (y—Mean; SD)	46.3 ± 14
Gender (F/M)	430/344
Current Smoker	152 (19.7)
Ethicity (N—%)	
Caucasian	719 (92.9)
Asian	34 (4.6)
Black	14 (1.8)
Sub-Saharan	4 (0.5)
North America	3 (0.4)
South America	0
Oceania	0
Comorbidities (N—%)	
Hypertension	141 (18.2)
Gastroesophageal disorders	88 (11.4)
Diabetes	84 (10.8)
Heart disorders	70 (9)
Asthma	66 (8.5)
Chronic renal failure	52 (6.7)
Chronic pulmonary disease	50 (6.5)
Depression	35 (4.5)
Neurological disorders	23 (3)
Liver failure	14 (1.8)
General Symptoms (N—%)	
Asthenia, malaise or confusion	475 (61.4)
Headache	348 (45)
Anorexia	306 (39.5)
Cough	302 (39.1)
Myalgia	302 (39.1)
Dyspnea	235 (30.3)
Fever (>38C)	235 (30.3)
Diarrhea	219 (28.3)
Arthralgia	213 (27.5)
Chest pain	175 (22.6)
Sticky mucus/phlegm	157 (20.3)
Abdominal pain	131 (16.9)
Nausea, vomiting	116 (15)
Clinical severity (N—%)	
Asymptomatic	111 (14.3)
Mild-to-moderate	519 (67.1)
Severe-to-critical	104 (13.4)
Missing data	40 (5.2)

Abbreviations: F/M: female/male; N: number; SD: standard deviation; y: year.

**Table 2:** Otolaryngological symptom severity of COVID-19 patients.

Otolaryngological Symptom Severity						
	Prevalence	Absent (0)	Mild (1)	Moderate (2)	Severe (3)	Very Severe (4)
Nasal obstruction	39.7	467 (60.3)	147 (19)	109 (14.1)	42 (5.4)	9 (1.2)
Rhinorrhea	37.5	484 (62.5)	164 (21.1)	68 (8.9)	47 (6.1)	11 (1.4)
Postnasal drip	30.6	537 (69.4)	105 (13.6)	79 (10.2)	45 (5.8)	8 (1)
Dysphonia	21.2	610 (78.8)	81 (10.5)	42 (5.4)	29 (3.7)	12 (1.5)
Sore throat	10.9	629 (81.3)	84 (10.8)	27 (3.5)	24 (3.1)	10 (1.3)
Ear pain	17.7	637 (82.3)	94 (12.1)	24 (3.1)	16 (2.1)	3 (0.4)
Face pain/heaviness	16	650 (84)	70 (9)	38 (4.9)	13 (1.7)	3 (0.4)
Dysphagia	10.9	690 (89.1)	32 (4.1)	35 (4.5)	10 (1.3)	7 (0.9)

**Table 3:** Olfactory outcomes of COVID-19 patients.

Self Reported OD (N = 774)	Number (%)
Olfactory dysfunction	451 (58.3)
Hyposmia	221 (28.5)
Anosmia	230 (29.7)
Parosmia	204 (26.4)
Phantosmia	105 (13.6)
No olfactory dysfunction	323 (41.7)
Psychophysical Olfactory Tests (N = 774)	
Normosmic	293 (37.8)
Hyposmic	280 (36.2)
Anosmic	201 (26)
Psychophysical Olfactory Tests (N = 774)	
Before the other symptoms	70 (15.5)
Concomitant with other symptoms	157 (34.8)
After the other symptoms	194 (43)
Did not remember/Missing data	30 (6.6)

Abbreviations: OD: olfactory dysfunction.

## Data Availability

L.A.V.; L.A. and J.R.L. had full access to all of the data in the study and take responsibility for the integrity of the data and the accuracy of the data analysis.

## Supplementary Materials

The following are available online at https://www.mdpi.com/2076-0817/10/1/62/s1, Table S1: Olfactory, clinical and epidemiological outcome correlations. Table S2: Cross-tab analysis results.
