# Supplementary material for: Psychophysical Evaluation of the Olfactory Function: European Multicenter Study on 774 COVID-19 Patients
Source: Pathogens. 2021 Jan 12;10(1):62. doi: 10.3390/pathogens10010062 (PMC7827350; doi:10.3390/pathogens10010062)
Supplement: Supplementary file 1 [file pathogens-10-00062-s001.pdf]

Table 1. Olfactory, clinical and epidemiological outcome correlations.

Variable	P-value
Gender	0.177
Age	0.362
Clinical severity	**0.038**
Diabetes	0.146
Hypertension	0.849
Gastroesophageal disorders	**0.003**
Asthma	0.855
Heart disorders	0.275
Smoker	0.258
Fever	**0.011**
Cough	0.223
Chest pain	**0.002**
Anorexia	0.159
Sticky mucus/phlegm	**0.035**
Arthralgia	0.219
Myalgia	0.230
Diarrhea	0.104
Abdominal pain	0.054
Nausea, vomiting	0.065
Headache	0.708
Asthenia, malaise or confusion	0.227
Dyspnea	**0.027**
Nasal Obstruction	0.184
Rhinorrhea	0.279
Postnasal drip	0.287
Sore throat	0.203

Ear pain	0.961
Face pain	0.355
Dysphagia	0.276
Dysphonia	**0.014**

Table S2: Cross-tab analysis results

**Variable**	**Z**	**OR**	**95% Confidence interval**	**P-value**
Clinical severity				**0.038**
Hyposmia	0.297	0.931	0.582 - 1.490	0.766
Anosmia	2.425	0.484	0.269 - 0.87	**0.015**
Anosmia or hyposmia	1.467	0.726	0.474 - 1.114	0.142
Gastroesophageal disorders				**0.003**
Hyposmia	2.426	1.86	1.127 - 3.072	**0.015**
Anosmia	3.345	2.425	1.443 - 4.975	**< 0.001**
Anosmia or hyposmia	2.228	1.67	1.064 - 2.623	**0.003**
Fever				**0.011**
Hyposmia	2.614	1.61	1.126 - 2.302	**0.009**
Anosmia	0.083	0.983	0.659 - 1.467	0.934
Anosmia or hyposmia	1.651	1.31	0.951 - 1.806	0.1
Chest pain				**0.002**
Hyposmia	2.141	1.516	1.036 - 2.22	**0.032**
Anosmia	1.453	0.705	0.441 - 1.129	0.934
Anosmia or hyposmia	0.752	1.144	0.806 - 1.625	0.452
Sticky mucus/phlegm				**0.035**

Hyposmia	2.535	1.701	1.128 - 2.566	**0.011**
Anosmia	0.865	1.229	0.77 - 1.96	0.387
Anosmia or hyposmia	2.099	1.496	1.027 - 2.178	**0.036**
<u>Dyspnea</u>				**0.027**
Hyposmia	0.114	1.02	0.720 - 1.446	0.909
Anosmia	2.373	0.609	0.404 - 0.917	**0.018**
Anosmia or hyposmia	1.134	0.834	0.609 - 1.141	**0.036**
<u>Dysphonia</u>				**0.014**
Hyposmia	0.990	1.215	0.826 - 1.789	0.322
Anosmia	2.058	0.603	0.373 - 0.976	**0.034**
Anosmia or hyposmia	0.348	0.939	0.659 - 1.338	0.728

Table S2 footnotes: Abbreviations: OR: odds ratio
